# From Nothing to Something: A Feasibility Pilot of a Sustainable, 3D-Printed, Social Enterprise Occupational Therapy Intervention for Severe Mental Illness

**DOI:** 10.1192/j.eurpsy.2026.11707

**Published:** 2026-07-31

**Authors:** P. Argitis, M. Demetriou, M. Peyioti, C. Tsamis, S. Moschat, M. Routsi, S. Bellos

**Affiliations:** 1 Psychiatric, General Hospital of Corfu, Corfu, Greece; 2 Psychiatric, General Hospital of Corfu, Corfu, Cyprus; 3 Psychiatric, University Hospital of Alexandroupoli, Corfu; 4 Psychiatric, General Hospital of Filiates, Igoumenitsa; 5 Psychiatric, University Hospital of Ioannina, Ioannina, Greece

## Abstract

**Introduction:**

Psychosocial rehabilitation for individuals with severe mental illness (SMI) often faces challenges in sustaining engagement, building transferable skills, and enhancing purpose and self-efficacy. Conventional occupational therapy may not always provide tangible or empowering experiences. There is a need for creative, interdisciplinary interventions that meet therapeutic goals while addressing real-world concerns like sustainability and meaningful work.

*From Nothing to Something* is a novel program integrating sustainable practice (upcycling PET), digital fabrication (3D printing), and social enterprise into rehabilitation. Participants transform discarded plastic into co-designed, marketable 3D products, creating therapeutic opportunities that are innovative and socially relevant.

**Objectives:**

This pilot study aims to test the feasibility and acceptability of the *From Nothing to Something* protocol for adults with SMI. Secondary aims include assessing preliminary impacts on therapeutic engagement, acquisition of vocational and cognitive skills, and psychosocial outcomes such as self-esteem and motivation. Additionally the study seeks to explore participant experiences to refine the intervention and identify core therapeutic mechanisms that could inform future program development and scaling.

**Methods:**

Quantitative data collection will include process logs, attendance records, staff-rated participation scales, a custom-developed vocational skills checklist, and validated psychometric instruments such as the Rosenberg Self-Esteem Scale and an adapted Interest and Motivation Checklist, administered pre- and post-intervention. Qualitative data will be gathered via participant interviews and focus groups to capture experiential feedback. Quantitative analysis will use descriptive statistics and pre-post comparisons (paired t-tests or Wilcoxon signed-rank tests), while qualitative data will undergo thematic analysis.

**Results:**

We hypothesize high feasibility and acceptability, as indicated by attendance, participation levels, and positive participant feedback. Improvements are anticipated in self-esteem motivation, and practical skill development. Qualitative findings are expected to provide insight into the intervention’s therapeutic impact and areas for refinement. These results will inform effect size estimates and guide the design of a future controlled efficacy trial.

**Conclusions:**

From Nothing to Something offers a promising, interdisciplinary model for occupational therapy that is both sustainable and empowering. By blending environmental consciousness with digital fabrication and social collaboration, this intervention may provide a replicable and impactful approach to enhancing recovery and community integration for individuals with SMI. This feasibility study represents a critical step toward a larger, methodologically robust trial.

**Disclosure of Interest:**

None Declared

